# The value of quantitative magnetic resonance imaging signal intensity in distinguishing between spinal meningiomas and schwannomas

**DOI:** 10.7150/ijms.73319

**Published:** 2022-06-21

**Authors:** Nguyen Duy Hung, Le Thanh Dung, Dang Khanh Huyen, Ngo Quang Duy, Dong-Van He, Nguyen Minh Duc

**Affiliations:** 1 Department of Radiology, Hanoi Medical University, Hanoi, Vietnam.; 2 Department of Radiology, Viet Duc Hospital, Hanoi, Vietnam.; 3 Department of Radiology, VNU University of Medicine and Pharmacy, Vietnam National University, Hanoi, Vietnam.; 4 Department of Radiology, Ha Giang General Hospital, Ha Giang, Vietnam.; 5 Department of Neurosurgery, Viet Duc Hospital, Hanoi, Vietnam.; 6 Department of Radiology, Pham Ngoc Thach University of Medicine, Ho Chi Minh City, Vietnam.

**Keywords:** spinal tumor, meningioma, schwannoma, quantitative measurement, magnetic resonance imaging

## Abstract

**Background:** Prior studies have suggested a number of the subjective visual characteristics that help distinguish between spinal meningiomas and schwannomas on magnetic resonance imaging and computed tomography; however, objective quantification of the signal intensity can be useful information. This study assessed whether quantitative magnetic resonance imaging (MRI) signal intensity (SI) measurements could distinguish intradural-extramedullary schwannomas from meningiomas.

**Methods:** From July 2019 to September 2021, 54 patients with intradural-extramedullary tumors (37 meningiomas and 17 schwannomas) underwent surgery, and tumors were verified pathologically. Defined regions of interest were used to quantify SI values on T1- (T1W) and T2-weighted images (T2W). Receiver operating characteristic curve analysis was used to obtain cutoff values and calculate the area under the curve (AUC), sensitivity (SE), specificity (SP), positive predictive value (PPV), and negative predictive value (NPV).

**Results:** Both Maximum (T2_max_) and mean (T2_mean_) T2W SI values demonstrated outstanding (AUC: 0.91) abilities to differentiate meningiomas from schwannomas with Se, Sp, PPV, and NPV values of 94.6%, 70.6%, 87.5%, and 85.7%, respectively, for T2_max_ and 81.1%, 88.2%, 93.8%, and 68.2% for T2_mean_. The maximum SI value on contrast-enhanced T1W (T1CE_max_) and the T2W tumor: fat SI ratio (rTF) demonstrated acceptable abilities (AUC: 0.73 and 0.79, respectively) to differentiate meningiomas from schwannomas with Se, Sp, PPV, and NPV values of 94.6%, 70.6%, 87.5%, and 85.7%, respectively, for T1CE_max_ and 81.1%, 88.2%, 93.8%, and 68.2% for rTF.

**Conclusions:** Quantitative SI values (T2_max_, T2_mean,_ T2_min_, T1CE_max_, rTF) can be used to differentiate intradural-extramedullary schwannomas from meningiomas.

## Introduction

Spinal schwannomas and meningiomas are two of the most common types of intradural-extramedullary tumors (representing 55%-90% of all intradural-extramedullary tumors) 1-3. Surgical resection is widely considered the best treatment option for both tumor types 4,5. However, each tumor type requires a different surgical approach depending on its origins. Spinal schwannoma resection typically requires the removal of involved nerve roots, whereas the surgical resection of a spinal meningioma requires total dural base excision due to the high risk of local recurrence 5,6. Therefore, preoperative histopathological predictions of the tumor type can be highly beneficial and contribute to the appropriate selection of surgical methods, treatment planning, and the prediction of prognosis.

Magnetic resonance imaging (MRI) of the spine combined with contrast injection is one of the best modalities for evaluating intraspinal tumors. Some recognized advantages of MRI assessment include its non-invasive nature and the ability to obtain high-quality images without radiation exposure 6,8. Although pathology remains the gold standard for final tumor diagnoses, the benefits of preoperative, MRI-based histopathologic type predictions are becoming more widely acknowledged and accepted 2,9-12. However, overlapping radiographic characteristics on MRI between schwannoma and meningioma can lead to misdiagnosis. According to Satoshi *et al.* 13, the sensitivity (Se), specificity (Sp), and accuracy when using T2-weighted images (T2W) for the differential diagnosis between spinal schwannomas and meningiomas were 95%-100%, 26%-42%, and 69%-73%, respectively, whereas the use of post-contrast T1-weighted images (T1W) resulted in Se, Sp, and accuracy values of 96%-100%, 56%-58%, and 81%-82%, respectively.

Although tremendous amounts of qualitative research exist regarding the differentiation of intradural-extramedullary schwannomas from meningiomas using MRI signals, the qualitative features used to differentiate these 2 tumor groups have typically relied on the experience of the researchers 2,9,11,14. We conducted a quantitative study of spinal schwannomas and meningiomas based on pre-and post-contrast MRI sequences, aiming to identify methods for differentiating between these two entities using conventional MRI assessments.

## Methods

### Study population

This retrospective study was conducted, including 54 patients with intradural-extramedullary spinal tumors who underwent surgical resection between July 2019 and September 2021. All tumors were verified to be either schwannoma or meningioma by pathology reports. Spinal MRI was performed preoperatively (9 cervical spines, 28 thoracic spines, 16 lumbar spines, and 1 sacral spine) at Viet Duc Hospital, Hanoi, Vietnam. All pathological results were evaluated by a pathologist with 20 years of experience in neuropathology. Ethical approval was received from the institutional ethics committee (Ref: 2682/QĐ-ĐHYHN, 20th July 2021), and the necessity of obtaining informed consent from the patients was waived due to the retrospective nature of this study.

### MRI technique

Our study was performed on Siemens 1.5T Magnetom Essenza (Siemens Medical Systems, Erlangen, Germany) or Philips Ingenia 1.5T (Philips Medical Systems, Netherlands) using a series of MRI sequences, including pre-and post-contrast sagittal T1W and sagittal and axial T2W. All images were obtained using a standard protocol (Table 1). The contrast agent was gadolinium-diethylene triamine pentaacetic acid (Gd-DTPA), which was administered intravenously at 0.2 ml/kg body weight.

### Image analysis

All MRI images were stored on the INFINITT PACS system (INFINITT Healthcare, South Korea) and were retrospectively analyzed by a radiologist with more than 10 years of experience in neuroradiology who was blinded to the pathological results.

**Figure 1:**
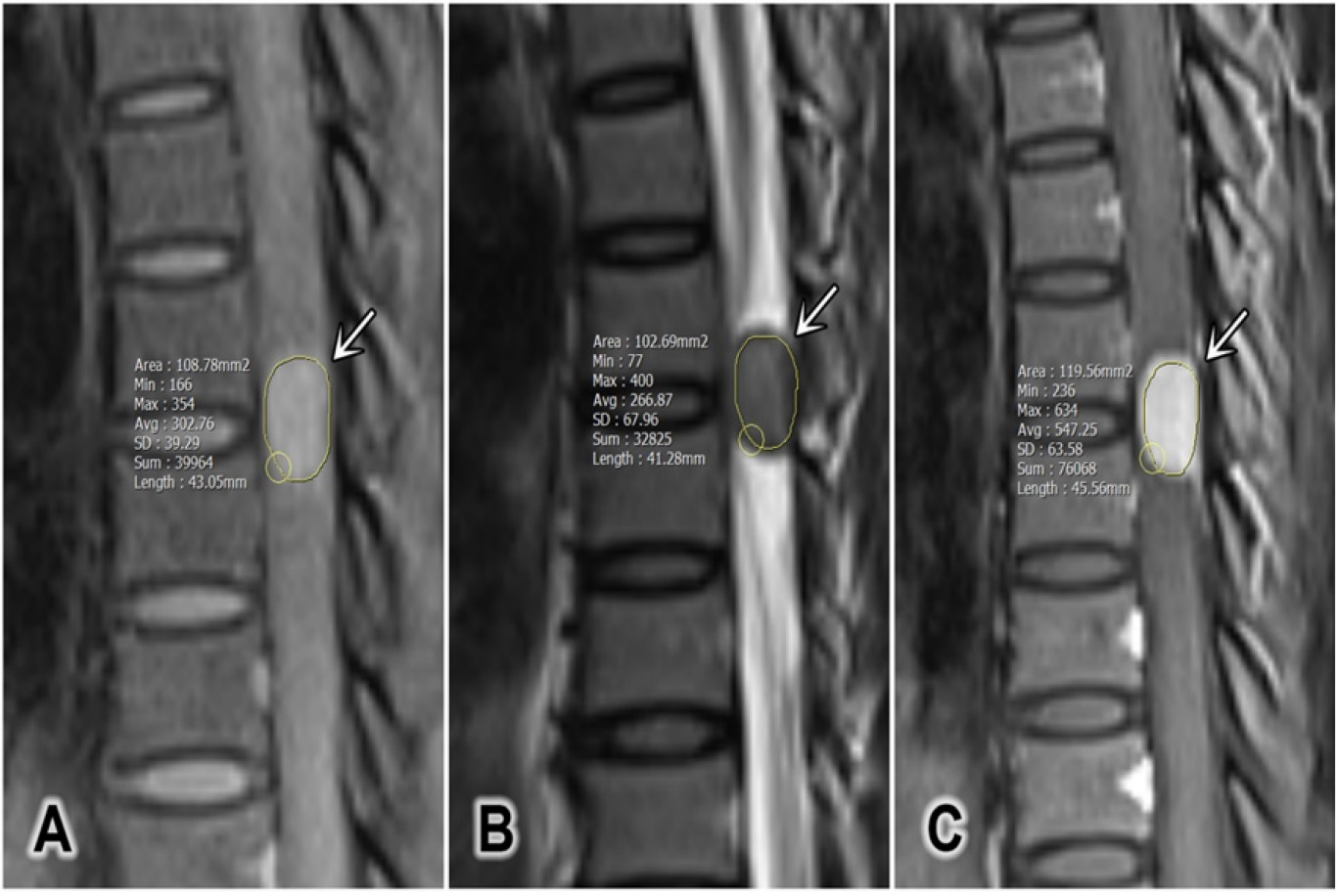
A representative region of interest defined for a tumor (white arrows). (A) Sagittal T1-weighted image, (B) sagittal T2-weighted image, and (C) sagittal contrast-enhanced T1-weighted fat-saturated image.

**Figure 2:**
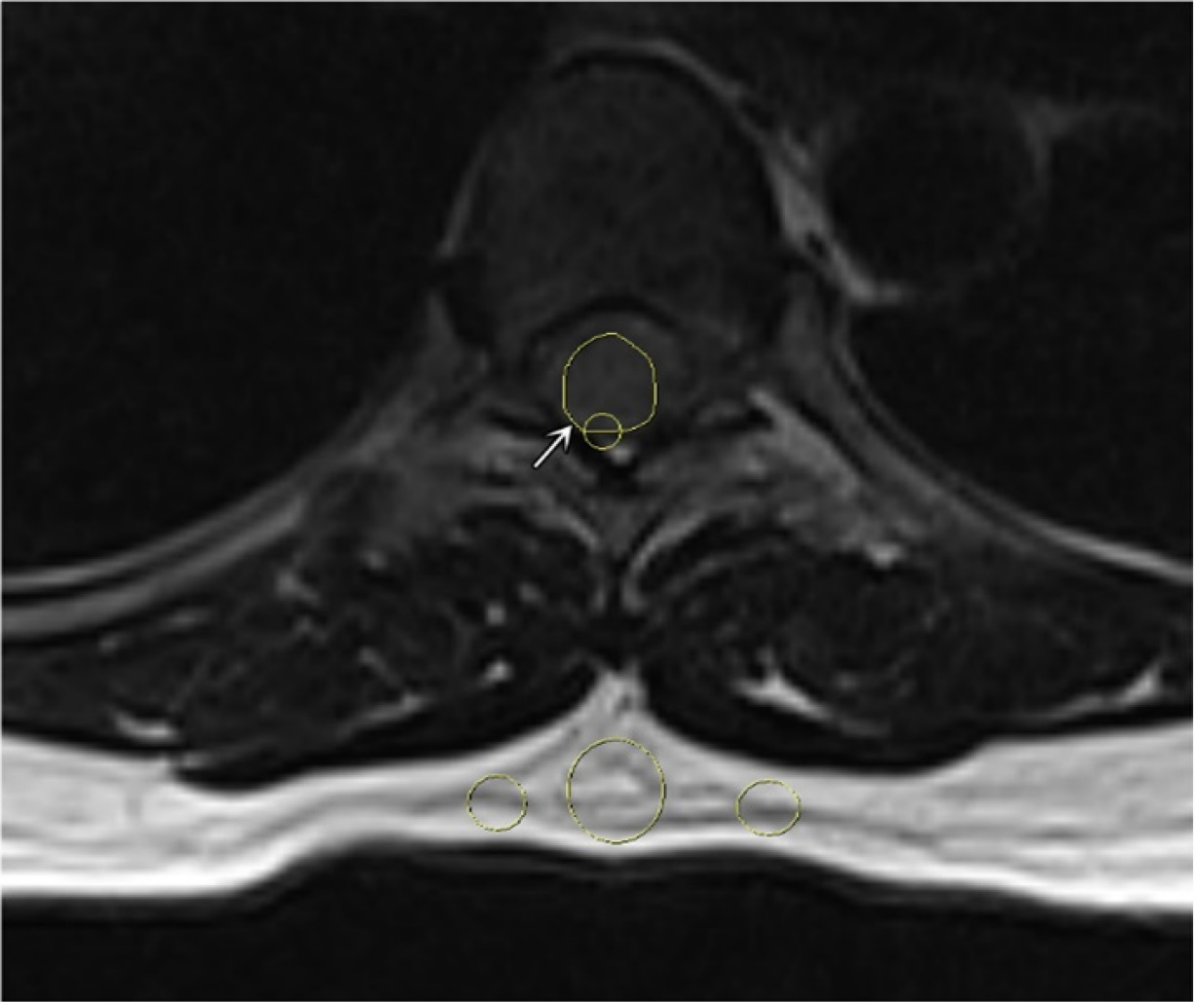
A representative region of interest on the tumor (white arrow) and 3 subcutaneous regions on axial T2-weighted image.

The tumor size was defined as the average of the anterior-posterior diameter and the greatest horizontal diameter on a single T2W slice 2. The longitudinal spinal location of the tumor was defined as cervical, thoracic, lumbar, or sacral. The horizontal location of the tumor was defined as anterior, posterior, or lateral. The signal intensity (SI) of the tumor on T2W was measured, and maximum (T2_max_), minimum (T2_min_), and mean (T2_mean_) values were recorded. Pre-contrast and contrast-enhanced T1W (T1CE) SI values were measured, and the maximum (T1_max_ and T1CE_max_), minimum (T1_min_ and T1CE_min_), and mean (T1_mean_ and T1CE_mean_) values were recorded in defined regions of interest (ROIs; Figure 1). The ROIs were hand-drawn and set with surrounding tumors on sagittal plane on T1W, T2W and T1CE to obtain the similar size of the ROI and to compare the tumor components on different sequences. At the largest area of the tumor, the ROI should cover at least two-thirds of the tumor diameter and should not contain any other surrounding structures (cerebral spinal fluid, bone, or ligamentum flavum). The SI for fat (SI fat) was determined by obtaining the mean value from three circular regions (covering 20-60 mm^2^) on the posterior side of the spinous process and both side pedicles. All of these values should be measured on the same slice, corresponding with the region in which the tumor size is the largest (Figure 2) 15. The tumor to fat SI ratio on T2W (rTF) was calculated as SI tumor/SI fat.

### Statistical analysis

The data were analyzed using SPSS 20.0 software (IBM Corp, Armonk, New York, United States). Quantitative variables are reported as medians and interquartile ranges due to non-standard distributions. Qualitative variables are reported as absolute numbers and percentages. The Mann-Whitney U test was used to test significant differences. Variables with normal distribution were tested with the Kolmogorov-Smirnov test. Differences between characteristics were analyzed using the Chi-square test and Fisher's exact test. P-values <0.05 were considered significant.

Receiver operating characteristic (ROC) curve analyses were performed to determine the cutoff values for distinguishing meningiomas from schwannomas. The area under the curve (AUC), Se, Sp, positive predictive value (PPV), and negative predictive value (NPV) were calculated.

## Results

### Patient characteristics

The patient characteristics according to tumor type are shown in Table 2. Among 54 patients, 37 cases were confirmed to be schwannomas, and 17 cases were meningiomas. Ages ranged from 18 to 83 years (mean: 50.9 years), including more women than men, with a female to male ratio of 1.84 to 1.

The mean age of the meningioma group was significantly higher than that of the schwannoma group. However, the schwannoma group showed significantly larger tumor sizes and a male predominance. Schwannomas were significantly more likely to be located at the cervical and lumbar spine on the longitudinal axis and in a lateral position on the horizontal axis than in other spinal locations. No significant differences were observed between the two tumor types for occurrence in women, thoracic or sacral spinal locations, or anterior and posterior locations.

### Signal intensity on MRI

**Figure 3:**
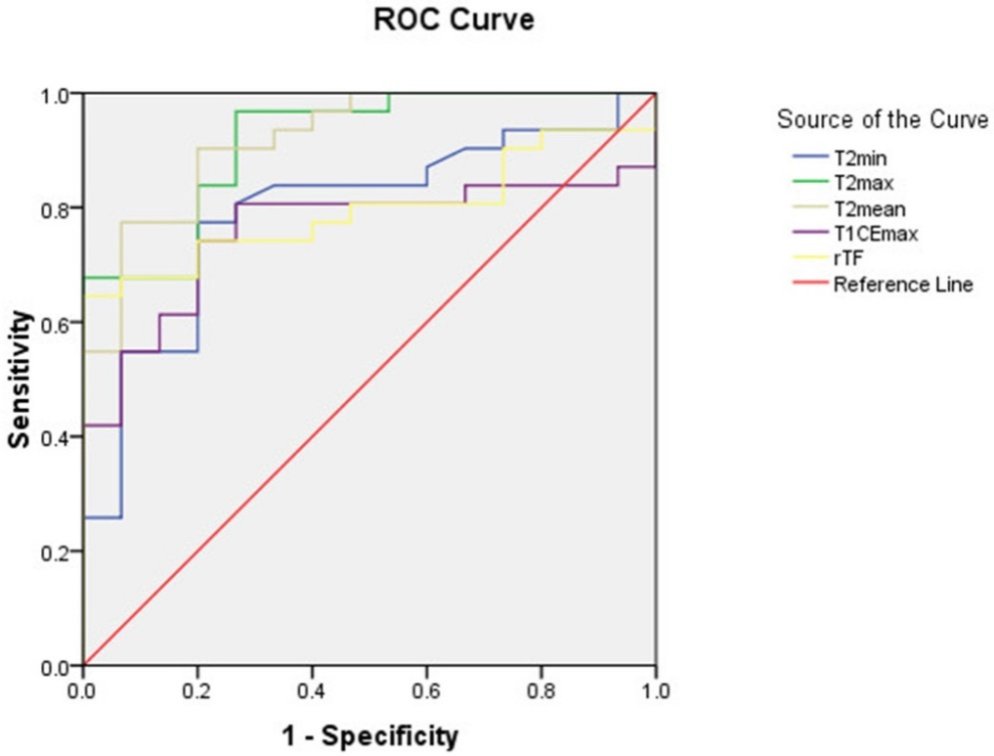
The receiver operating characteristic (ROC) curves for the minimum (T2_min_), maximum (T2_max_), and mean (T2_mean_) signal intensity values on T2-weighted images, the maximum signal intensity value on contrast-enhanced T1-weighted images (T1CE_max_), and the tumor to fat signal intensity ratio (rTF). *T2_min_, minimum signal intensity value on T2-weighted image (T2W); T2_max_, maximum signal intensity value on T2W; T2_mean_, mean signal intensity value on T2W; T1CE_max_, maximum signal intensity value on contrast-enhanced T1-weighted image; rTF: tumor to fat signal intensity ratio on T2W.*

As shown in table 3, T2_max_, T2_min_, T2_mean_, T1CE_max_, and rTF values for schwannomas were significantly higher than those for meningiomas. ROC curve analyses were performed for these parameters (Figure 3), and the diagnostic performances of these parameters are described in Table 4.

T2_max_ and T2_mean_ were outstanding for diagnosis (AUC > 0.9) with values for Se, Sp, PPV, and NPV of 94.6%, 70.6%, 87.5%, and 85.7%, respectively, for T2_max_, and 81.1%, 88.2%, 93.8%, and 68.2%, respectively, for T2_mean_ for differentiating between spinal meningiomas and schwannomas. T2_min_, T1CE_max_, and rTF were considered acceptable for diagnosis (0.7 < AUC < 0.8).

## Discussion

According to Hirano and his team, schwannomas and meningiomas are the two most common spinal intradural-extramedullary tumors, comprising 68.2% and 20.2% of all spinal intradural-extramedullary tumors, respectively 3. Based on their epidemiology, the differential diagnosis between meningiomas and schwannomas should be considered when diagnosing spinal intradural-extramedullary tumors. Although these tumors are generally benign, with slow progression, and rarely transform into malignancies, the associated symptoms often affect patients' quality of life 16,17. Surgery is typically the best choice of treatment for both tumor types, but the surgical approaches required for treatment are significantly different 6. An accurate preoperative radiographic diagnosis can contribute to the selection of an optimal treatment plan and improve prognosis 18. Spinal MRI is the recommended modality for the evaluation of spinal neoplasms 7. Despite several qualitative studies focused on differentiating between schwannoma and meningioma based on MRI and computed tomography results 2,9,11,14, to our knowledge, this is the first study to examine the quantitative differences of these two entities on T1W pre- and post-contrast and T2W.

The age of patients diagnosed with meningiomas in our study (median 61 years, 95%, CI: 41-83 years) was significantly higher than those diagnosed with schwannomas (median 49 years, 95% CI: 18-86 years), which is similar to study reported by Iwata *et al.* 14, in which the mean age of patients with meningioma was 68 years, ranging from 39 to 85 years, and the mean age of patients with schwannoma was 56.2 years, ranging from 18 to 84 years. Furthermore, the study by Lee *et al.* 10 reported a mean age for the meningioma group of 59.7 years, whereas those with schwannomas had a mean age of 47.6 years. Previous studies have predominantly agreed that the mean age of patients with schwannomas is significantly higher than that of patients with meningiomas. By contrast, a study by Zhai *et al.*, published in 2019 2, reported no significant difference between the mean ages of these two groups.

In this study, the tumor size of schwannomas (median 11.9; 95% CI: 7.7-59.6) was significantly larger than that of meningiomas (median 9.73; 95% CI: 6.5-23.9). The study by Lee *et al.* 10 also reported a similar result. Additionally, our study found that men were significantly more likely to be diagnosed with schwannoma than meningioma, and schwannomas were significantly more likely to be located in the cervical and lumbar spine and in a lateral position on the horizontal axis. Similar findings were reported by Zhai *et al.* 2, who reported a male predominance among schwannoma patients, with the most common lesion locations being the lumbar spine and the lateral posterior segment. However, meningiomas are more common than schwannomas in the thoracic region and in anterior, anterolateral segments. In 2005, Verdelhan *et al.* 11 reported that meningiomas were predominately located at the thoracic spine, whereas schwannomas were predominately located at the lumbar spine; however, no significant differences were reported for the horizontal axis.

According to previous studies, the SI on pre-contrast T1W was generally iso-intense or hypo-intense for both tumor types 6,12,19-21. Liu *et al.* 9 and Verdelhan *et al.*^11^ reported that 100% of schwannomas and meningiomas were iso-intense on pre-contrast T1W, with no qualitative differences. Our quantitative examination found similar results, with no significant differences between the two tumor types for T1_min_, T1_max_, and T1_mean_ values. By contrast, the study by Zhai *et al.* showed a lower SI on pre-contrast T1W for schwannomas compared with meningiomas, with diagnostic values for Sp, Se, PPV, and NPV of 53.8%, 94.3%, 94.9%, and 51%, respectively.

According to the present study, the quantitative T2_max_, T2_min_, and T2_mean_ values for schwannomas are significantly higher than those for meningiomas, which is similar to the results reported by studies examining the qualitative differences in SI on T2W for these two tumor types. Schwannomas presented with a heterogeneously hyperintense signal on T2W in the study reported by Verdelhan *et al.* 11. The studies by Iwata *et al.* 14, Zhai *et al.* 2, and Lee *et al.* 10 also reported significantly increased fluid signals on T2W in schwannomas are significantly higher than those of meningiomas. The fluid signals are indicated by signal intensity equal to that of the cerebrospinal fluid, exhibiting hypointense on T1WI, hyperintense on T2WI and show no enhancement after contrast administration. Research by Liu *et al.* 9 reported that 87% of schwannomas and 61.1% of meningiomas are hyperintense on T2W; thus, these two tumor types are difficult to distinguish on T2W alone. Our study demonstrated that quantitative measurements of SI on T2W could be useful for differentiating between meningiomas and schwannomas. Histopathologically, schwannomas can be subdivided into Antoni A and Antoni B types. The Antoni B type generally contains multiple cystic degeneration regions, which present as vivid fluid signals on T2W. According to Suzuki *et al.* 24 and Zhang *et al.* 25*,* spinal cystic meningiomas are extremely rare, and cystic meningiomas comprise only 2%-4% of all intracranial tumors.

The administration of gadolinium resulted in the vivid enhancement of both meningiomas and schwannomas 1,20,26. The report by Zhai *et al.* 2 suggest that no significant difference in SI can generally be detected on T1CE between the two tumor groups, but also show that schwannomas present with a ring-like enhancement pattern that was not visible in meningiomas. Our study also indicated quantitatively that there was no difference in T1CE_min_ and T1CE_mean_ value, but identified a significantly higher T1CE_max_ value for schwannomas compared with meningiomas, which can be explained by differences in gap junctions between these two groups. The gap junctions of schwannomas are typically short, straight, and directly linked to the extracellular space. By contrast, the gap junctions of meningioma feature zigzagged shapes composed of neoplastic cells 27,28. Breger *et al.* 29 studied the signal intensity value on contrast-enhanced T1-weighted image of benign extra-axial tumors on the brain, the results also show that the signal values on T1W after injection of schwannomas is higher than meningiomas. Research by Ota *et al.* 30 reported that differentiation between meningiomas and schwannomas in the cerebellopontine angle and jugular foramen can be relied on some parameters on dynamic-contrast enhanced imaging (DCE-MRI), with significant differences between these two tumor types for the fractional blood plasma volume (Vp) and extracellular extravascular space (Ktrans) (p < 0.001, <0.001, respectively; and AUC of Vp values > 0.9). The report by Meng *et al.* 31 assessed spinal tumor vascularity on DCE-MRI and showed that it is an accurate technique. However, to our knowledge, there are no studies on distinguish between spinal meningiomas and schwannomas on DCE-MRI.

Many previous studies have discussed the value of rTF 2,9,12,14. Takashima *et al.* 15 recommended using subcutaneous fat, which is easier and more consistent than bone marrow fat or muscular fat. Our study found a similar result as that reported by Takashima *et al.,* suggesting a significantly higher rTF for schwannomas than for meningiomas. The AUC for rTF, the cutoff value, and the Se and Sp values were 0.79, 0.63, 64.5%, and 100%, respectively, in our study and 0.78, 0.42, 80%, and 70%-75% in the study by Takashima *et al.* 15.

To our knowledge, artificial intelligence (AI) has been studied in diagnosis, prognosis, outcome prediction following spinal surgery, biomechanical assessments of spinal diseases such as scoliosis spinal deformity, spinal osteoarthritis, spinal cord tumors… 32,33. However, there have been no studies about AI in differentiating between spinal intradural-extramedullary schwannomas and meningiomas. We believe that our results can support a reference data for the future development of AI about intradural-extramedullary tumors.

This study has several limitations. First, the small sample size could introduce bias into the evaluation of diagnostic performance. Second, a single observer obtained all measurements. Third, in this study, we only aimed to focus on quantitative magnetic resonance imaging signal intensity; thus, it lacked the morphological information of tumors. Therefore, additional studies with larger sample size and more observers will likely improve the accuracy of our findings. In further study, a model for discriminating between spinal meningiomas and schwannomas combining morphological information and quantitative magnetic resonance imaging signal intensity can enhance diagnostic efficacy.

## Conclusion

The quantitative evaluation of SI values on MRI can be effective for differentiating between spinal schwannomas and meningiomas, with T2_max_, T2_min_, and T2_mean_ serving as the most valuable parameters.

## Figures and Tables

**Table 1:** MRI standard protocol.

Parameters	T1W	T2W	T1CE
Slice thickness (mm)	4	4	4
GAP (mm)	1	1	1
NEX	2	2	2
FOV (mm)	160-250	160-250	160-250
Matrix	256 × 256	256 × 256	256 × 256
TR (ms)	600-700	2000-3000	600-700
TE (ms)	20-30	90-100	20-30

MRI: Magnetic resonance imaging; FOV: Field of View; TR: repetition time; TE: echo time; T1W: T1-weighted image; T2W: T2-weighted image; T1CE: contrast-enhanced T1-weighted image.

**Table 2:** Patient clinical characteristics

Patient characteristics		Tumor		P-value
		Meningioma (n = 17)	Schwannoma (n = 37)	
Age (y)		61 (95% CI, 41-83)	49 (95% CI, 18-68)	0.00*
Sex, n (%)	Male	1 (5.9%)	18 (48.65%)	0.00*
	Female	16 (94.1%)	19 (51.4%)	0.74
Longitudinal location, n (%)	Cervical	1 (5.9%)	8 (21.6%)	0.04*
	Thoracic	14 (82.4%)	14 (37.8%)	1.00
	Lumbar	2 (11.8%)	14 (37.8%)	0.004*
	Sacral	0	1 (2.7%)	
Horizontal location, n (%)	Anterior	7 (41.2%)	6 (16.2%)	1.00
	Lateral	10 (58.8%)	26 (70.3%)	0.01*
	Posterior	0	5 (13.5%)	0.06
Size (mm)		9.73 (95% CI, 6.5-23.9)	11.9 (95% CI, 7.7-59.6)	0.039*

*Statistical analysis demonstrated a significant difference with a 95% confidence interval (95% CI) using binomial tests (p < 0.05). 95% CI: 95% confidence interval.

**Table 3:** Tumor characteristics on MRI.

Characteristics		Tumor		P-value
		Meningioma (n = 17)	Schwannoma (n = 37)	
Signal intensity on pre-contrast T1W	T1_min_	169 (95% CI, 81-573)	233 (95% CI, 51-802)	0.099
	T1_max_	287 (95% CI, 185-762)	397 (95% CI, 174-1509)	0.139
	T1_mean_	241.9 (95% CI, 146.6-675.5)	332.3 (95% CI, 88.95-1030.4)	0.196
Signal intensity on T2W	T2_min_	190 (95% CI, 39-452)	307 (95% CI, 86-1179)	0.001*
	T2_max_	406 (95% CI, 286-822)	859 (95% CI, 377-2278)	0.000*
	T2_mean_	283.3 (95% CI, 184.5-658.5)	623.1 (95% CI, 286.4-1892.1)	0.000*
Signal intensity on contrast-enhanced T1W (T1CE)	T1CE_min_	274 (95% CI, 167-822)	371 (95% CI, 45-895)	0.479
	T1CE_max_	487 (95% CI, 361-1287)	930 (95% CI, 272-2718)	0.006*
	T1CE_mean_	414.2 (95% CI, 302.2-1001.9)	658.9 (95% CI, 177-1639.54)	0.107
rTF		0.38 (95% CI, 0.24-0.59)	0.81 (95% CI, 0.16-3.68)	0.001*

*Statistical analysis demonstrated significant difference with 95% confidence interval (95% CI) using the Mann-Whitney U test (p < 0.05). MRI, magnetic resonance imaging; T2_max_, maximum signal intensity value on T2-weighted image (T2W); T2_min_, minimum signal intensity value on T2W; T2_mean_, mean signal intensity value T2W; T1_max_, maximum signal intensity value on pre-contrast T1-weighted image (T1W); T1_min_, minimum signal intensity value on T1W; T1_mean_, mean signal intensity value T1W; T1CE_max_, maximum signal intensity value on T1CE; T1CE_min_, minimum signal intensity value on T1CE; T1CE_mean_, mean signal intensity value on T1CE; rTF: the tumor/fat T2W SI ratio.

**Table 4:** Cutoff value, AUC, Se, Sp, PPV, and NPV of the significant parameters assessed during differential diagnosis between schwannomas and meningiomas.

Characteristics	Cutoff	Se	Sp	PPV	NPV	AUC
T2_min_	≥228	78.4	76.5	87.9	61.9	0.79
T2_max_	≥500	94.6	70.6	87.5	85.7	0.91
T2_mean_	≥423.35	81.1	88.2	93.8	68.2	0.91
T1CE_max_	≥819.5	59.5	88.2	91.7	50	0.73
rTF	≥0.63	64.5	100	100	57.7	0.79

T2_min_, minimum signal intensity value on T2-weighted image (T2W); T2_max_, maximum signal intensity value on T2W; T2_mean_, mean signal intensity value on T2W; T1CE_max_, maximum signal intensity value on contrast-enhanced T1-weighted image; rTF: tumor to fat signal intensity ratio; AUC, area under the curve; Se, sensitivity; Sp, specificity; PPV, positive predictive value; NPV, negative predictive value.

## References

[1] Intradural spinal tumors (2021). current classification and MRI features. SpringerLink. Accessed April 6.

[2] Zhai X, Zhou M, Chen H (2019). Differentiation between intraspinal schwannoma and meningioma by MR characteristics and clinic features. *Radiol Med (Torino)*.

[3] Hirano K, Imagama S, Sato K (2012). Primary spinal cord tumors: review of 678 surgically treated patients in Japan. A multicenter study. *Eur Spine J*.

[4] Engelhard HH, Villano JL, Porter KR (2010). Clinical presentation, histology, and treatment in 430 patients with primary tumors of the spinal cord, spinal meninges, or cauda equina: Clinical article. *J Neurosurg Spine*.

[5] Ahn DK, Park HS, Choi DJ, Kim KS, Kim TW, Park SY (2009). The surgical treatment for spinal intradural extramedullary tumors. *Clin Orthop Surg*.

[6] Parsa AT, Lee J, Parney IF, Weinstein P, McCormick PC, Ames C (2004). Spinal cord and intradural-extraparenchymal spinal tumors: current best care practices and strategies. *J Neurooncol*.

[7] Gebauer GP, Farjoodi P, Sciubba DM (2008). Magnetic Resonance Imaging of Spine Tumors: Classification, Differential Diagnosis, and Spectrum of Disease. *JBJS*.

[8] Matsumoto S, Hasuo K, Uchino A (1993). MRI of intradural-extramedullary spinal neurinomas and meningiomas. *Clin Imaging*.

[9] Liu WC, Choi G, Lee SH (2009). Radiological findings of spinal schwannomas and meningiomas: focus on discrimination of two disease entities. *Eur Radiol*.

[10] Lee JH, Kim HS, Yoon YC, Cha MJ, Lee SH, Kim ES (2020). Differentiating between spinal schwannomas and meningiomas using MRI: A focus on cystic change. *PLOS ONE*.

[11] MR imaging features of spinal schwannomas and meningiomas - ScienceDirect Accessed April 9, 2021. https://www.sciencedirect.com/science/article/pii/S0150986105830214?casa_token=Z9nVdMuPXFUAAAAA:CE8r1ZkxaZyIUhXRGQtf5Nn7lfOKa44eVIR6Yptev7Z76Iv63d4AoxyDoxhMFQk25jMQu74XE5M.

[12] Gu R, Liu JB, Zhang Q, Liu GY, Zhu QS (2014). MRI diagnosis of intradural extramedullary tumors. *J Cancer Res Ther*.

[13] Maki S, Furuya T, Horikoshi T (2020). A Deep Convolutional Neural Network With Performance Comparable to Radiologists for Differentiating Between Spinal Schwannoma and Meningioma. *Spine*.

[14] Iwata E, Shigematsu H, Yamamoto Y (2018). Preliminary algorithm for differential diagnosis between spinal meningioma and schwannoma using plain magnetic resonance imaging. *J Orthop Sci Off J Jpn Orthop Assoc*.

[15] Takashima H, Takebayashi T, Yoshimoto M (2018). Differentiating spinal intradural-extramedullary schwannoma from meningioma using MRI T2 weighted images. *Br J Radiol*.

[16] Grimm S, Chamberlain MC (2009). Adult primary spinal cord tumors. *Expert Rev Neurother*.

[17] Ozawa H, Onoda Y, Aizawa T, Nakamura T, Koakutsu T, Itoi E (2012). Natural history of intradural-extramedullary spinal cord tumors. *Acta Neurol Belg*.

[18] Nakamizo A, Suzuki SO, Shimogawa T (2012). Concurrent spinal nerve root schwannoma and meningioma mimicking single-component schwannoma. *Neuropathology*.

[19] Wu L, Yao N, Chen D, Deng X, Xu Y (2011). Preoperative diagnosis of intramedullary spinal schwannomas. *Neurol Med Chir (Tokyo)*.

[20] Soderlund KA, Smith AB, Rushing EJ, Smirniotopolous JG (2012). Radiologic-pathologic correlation of pediatric and adolescent spinal neoplasms: Part 2, Intradural extramedullary spinal neoplasms. *AJR Am J Roentgenol*.

[21] Intradural Extramedullary Spinal Neoplasms (2021). Radiologic-Pathologic Correlation. RadioGraphics. Accessed April 5.

[22] Wippold FJ, Lubner M, Perrin RJ, Lämmle M, Perry A (2007). Neuropathology for the neuroradiologist: Antoni A and Antoni B tissue patterns. *AJNR Am J Neuroradiol*.

[23] Ando K, Imagama S, Ito Z (2016). How do spinal schwannomas progress? The natural progression of spinal schwannomas on MRI. *J Neurosurg Spine*.

[24] Suzuki A, Nakamura H, Konishi S, Yamano Y (2002). Dumbbell-shaped meningioma with cystic degeneration in the thoracic spine: a case report. *Spine*.

[25] Zhang J, Chen Z he, Wang Z feng (2016). Epidural Cystic Spinal Meningioma. *Medicine (Baltimore)*.

[26] Parizel P, Baleriaux D, Rodesch G (1989). Gd-DTPA-enhanced MR imaging of spinal tumors. *Am J Roentgenol*.

[27] Kasantikul V, Glick AD, Netsky MG (1979). Light and electron microscopic observations of blood vessels in neurilemoma. *Arch Pathol Lab Med*.

[28] Watabe T, Azuma T (1989). T1 and T2 measurements of meningiomas and neuromas before and after Gd-DTPA. *AJNR Am J Neuroradiol*.

[29] Breger RK, Papke RA, Pojunas KW, Haughton VM, Williams AL, Daniels DL (1987). Benign extraaxial tumors: contrast enhancement with Gd-DTPA. *Radiology*.

[30] Ota Y, Liao E, Capizzano AA (2022). MR diffusion and dynamic-contrast enhanced imaging to distinguish meningioma, paraganglioma, and schwannoma in the cerebellopontine angle and jugular foramen. J Neuroimaging Off J Am Soc Neuroimaging.

[31] Meng XX, Zhang YQ, Liao HQ (2016). Dynamic contrast-enhanced MRI for the assessment of spinal tumor vascularity: correlation with angiography. Eur Spine J Off Publ Eur Spine Soc Eur Spinal Deform Soc Eur Sect Cerv Spine Res Soc.

[32] Azimi P, Yazdanian T, Benzel EC, Aghaei HN, Azhari S, Sadeghi S, Montazeri A (2020). A Review on the Use of Artificial Intelligence in Spinal Diseases. Asian Spine J.

[33] Lemay A, Gros C, Zhuo Z, Zhang J, Duan Y, Cohen-Adad J (2022). Multiclass spinal cord tumor segmentation on MRI with deep learning. 2020. arXiv:2012.12820 [cs, eess], Mar. 2021, Accessed: Mar. 10.

